# Hydroxyapatite Use in Spine Surgery—Molecular and Clinical Aspect

**DOI:** 10.3390/ma15082906

**Published:** 2022-04-15

**Authors:** Jakub Litak, Wojciech Czyzewski, Michał Szymoniuk, Bartlomiej Pastuszak, Joanna Litak, Grzegorz Litak, Cezary Grochowski, Mansur Rahnama-Hezavah, Piotr Kamieniak

**Affiliations:** 1 Department of Neurosurgery, Medical University of Lublin, Jaczewskiego 4, 20-090 Lublin, Poland; jakub.litak@gmail.com (J.L.); wojciech.w.czyzewski@gmail.com (W.C.); michmatsz@gmail.com (M.S.); pastuszak.bartlomiej@gmail.com (B.P.); pkamieniak@poczta.onet.pl (P.K.); 2 Department of Didactics and Medical Simulation, Medical University of Lublin, Chodzki 4, 20-090 Lublin, Poland; 3 Centrum Onkologii Ziemi Lubelskiej, Jaczewskiego 4, 20-090 Lublin, Poland; litak.joanna@gmail.com; 4 Department of Automation, Faculty of Mechanical Engineering, Lublin University of Technology, Nadbystrzycka 36, 20-618 Lublin, Poland; g.litak@pollub.pl; 5 Laboratory of Virtual Man, Medical University of Lublin, 20-439 Lublin, Poland; 6 Department of Dental Surgery, Medical University of Lublin, 20-081 Lublin, Poland; mansur.rahnama@umlub.pl

**Keywords:** HAp, hydroxyapatite, spine, surgery

## Abstract

Hydroxyapatite possesses desirable properties as a scaffold in tissue engineering: it is biocompatible at a site of implantation, and it is degradable to non-toxic products. Moreover, its porosity enables infiltration of cells, nutrients and waste products. The outcome of hydroxyapatite implantation highly depends on the extent of the host immune response. Authors emphasise major roles of the chemical, morphological and physical properties of the surface of biomaterial used. A number of techniques have been applied to transform the theoretical osteoconductive features of HAp into spinal fusion systems—from integration of HAp with autograft to synthetic intervertebral implants. The most popular uses of HAp in spine surgery include implants (ACDF), bone grafts in posterolateral lumbar fusion and transpedicular screws coating. In the past, autologous bone graft has been used as an intervertebral cage in ACDF. Due to the morbidity related to autograft harvesting from the iliac bone, a synthetic cage with osteoconductive material such as hydroxyapatite seems to be a good alternative. Regarding posterolateral lumbar fusion, it requires the graft to induce new bone growth and reinforce fusion between the vertebrae. Hydroxyapatite formulations have shown good results in that field. Moreover, the HAp coating has proven to be an efficient method of increasing screw fixation strength. It can decrease the risk of complications such as screw loosening after pedicle screw fixation in osteoporotic patients. The purpose of this literature review is to describe in vivo reaction to HAp implants and to summarise its current application in spine surgery.

## 1. Introduction

Hydroxyapatite (Ca_10_(PO_4_)_6_(OH)_2_, HAp) is the most significant inorganic component of teeth and bone tissue [1]. Due to their crystallographic and chemical similarity with human bone tissue, synthetic ceramics based on hydroxyapatites are commonly used in biomedical applications, such as dentistry and orthopaedics, including spine surgery [2,3].

The outcome of hydroxyapatite implantation highly depends on the extent of the host immune response [4,5]. This significantly affects tissue remodelling as well as wound healing processes caused by a surge of reactions taking place on the Hap–tissue interface that includes angiogenesis, activation of the fibroblast, as well as healing and remodelling of the matrix. A created immunological microenvironment stands behind a positive outcome of HAp integration [6,7]. Cells participating in host–cell response in embedding hydroxyapatite are, among others, mast cells, macrophages, neutrophils, and multinucleated giant cells [8]. Recruitment of monocytes and neutrophils and subsequent differentiation of monocytes to macrophages initialises the process [9]. The aforementioned immune cells not only produce ROS designed to eliminate the foreign body but also produce a span of cytokines and chemokines that stimulate fibroblasts, endothelial cells, and mesenchymal stem cells to create a new tissue [10,11,12,13,14].

Hydroxyapatite possesses desirable properties as a scaffold in tissue engineering: it is biocompatible at a site of implantation and degradable to non-toxic products. Moreover, its porosity enables the infiltration of cells, nutrients, and waste products [15].

The authors emphasise the major role of the chemical, morphological and physical properties of the surface of biomaterial used [16,17,18]. The modulated reactions include cell adhesion, formation of the foreign body giant cells, and protein absorption. Multiple papers proved the capability to stimulate osteoinduction, depending on the material’s texture [19,20,21,22,23,24]. Regarding the type of texture, MSC differentiates to osteoblasts accordingly [25,26]. Modifications of chemical structure also have a vast impact on immunoreactivity [27]. Moreover, the aging of HAp implants, including radiation exposure, has a significant influence on its clinical performance [28,29].

Hydroxyapatite itself varies in morphological and physicochemical features as solubility, crystallinity, granulometric distribution as well as shape and size of pores. In a study on animal models in a team led by da Freitas Costa, these differences did not have an impact on cellular response [30]. According to Sadowska et al., incubation of RAW murine cells with less porous calcium-deficient HAp (CD-HAp) generated the release of a decreased amount of pro-inflammatory cytokines [31].

Laquerriere et al. underlined various immune responses to the HAp particles’ features such as shape, size, or sintering temperature. Phagocytable spherical molecules increase expression as well as production by monocytes of TNF alpha and IL-6, in contrast to these non-phagocytable cells, which had an influence on neither. Moreover, needle-shaped HAp particles had the highest impact on TNF alpha, IL-6, and IL-10 production [32]. The degree of immune response in vivo and in vitro was analysed in regard to HAp size and morphology by Filipa Labre et al. In this study, the inflammatory response was prolonged in smaller needle-shaped HAp particles compared to other shapes of HAp particles [33].

Understanding the reactions evoked by incorporated hydroxyapatite seems pivotal in anticipation of its biocompatibility [34].

Due to the recent development of micro- and nanotechnology, a wide range of biomaterials also differ in immunomodulatory effects [6,35,36]. Micro- and nanotopography are the key factors in the induction of osteogenesis by hydroxyapatite [37].

The most popular uses of HAp in spine surgery include implants (ACDF), bone grafts in posterolateral lumbar fusion, and transpedicular screws coating.

In the past, autologous bone graft has been used as an intervertebral cage for ACDF. Due to the morbidity related to autograft harvesting from iliac bone, a synthetic cage with an osteoconductive material such as hydroxyapatite seems to be a good alternative [38]. Regarding posterolateral lumbar fusion, it requires a graft to induce new bone growth and reinforce fusion between vertebrae. Hydroxyapatite formulations have shown good results in that field [39,40,41].

Moreover, a HAp coating has proven to be an efficient method of increasing screw fixation strength. It can decrease the risk of complications such as screw loosening after pedicle screw fixation in osteoporotic patients [42,43].

This literature review describes in vivo immunologic reactions to HAp implants and summarises its current application in spine surgery.

## 2. Immunologic Reaction to Hydroxyapatite

### 2.1. Mast Cells, Cytokines, and Chemokines

The host recognises hydroxyapatite as a foreign body, which triggers cytokines and chemokines release [44]. Commonly, after this acute phase, the inflammatory state decreases, somatic cells proliferate and tissue remodelling occurs, which results in restoration of haemostasis. In place of unsuccessful resolution of a chronic phase that occurs, heralded by the fusion of macrophages, foreign body giant cell formation and encapsulation of the biomaterial takes place [45]. Degranulation of mast cells with histamine, IL-4, and IL-13 is known to be responsible for the foreign body inflammatory response. Subsequently, phagocytes, i.e., macrophages, are being recruited to adhere to the implant’s surface. This process is enhanced by the absorption of the host’s fibrinogen. Various adsorbed proteins including albumins, fibronectin, complement, gamma globulin, and vitronectin modulate the host immune response to hydroxyapatite. The degree of adsorption is highly dependent on HAp crystallinity [46], surface charge [47], and others [48]. After the acute phase of inflammation begins, the chronic phase is demarcated by the recruitment of mononuclear cells (lymphocytes and monocytes). 

### 2.2. Macrophage Recruitment

Extravasation and migration of monocytes/macrophages are induced by cytokines and chemokines as CXC, CC, C, and CX3C. Other particles, directing macrophages to the site of the foreign body, are TGF-β, PDGF, PF4, leukotriene, and IL-1 released by platelets and blood clots [49]. Macrophages themselves, as the site of biomaterial, release further PDGF, TNF-α, IL-6, G-CSF, and GM-CSF, with the latter attracting more macrophages. As reported by Mesters et al., differences in the HAp substrate’s microstructure, whether with micrometric plate-like or nanometric needle-like crystals, differentiate in a degree of macrophage proliferation and activation. Plate-like crystals are characterised by a higher velocity of proliferation, which is believed to be due to less pronounced depletion of Ca ions with cell medium after contact with C-HAp. A lower release of reactive oxygen species was also observed in needle-like substrates [10]. Many chemokines are released, including CCL2, CCL3, CCL4, CCL7, CCL8, and CCL13, which recruit macrophages in the biomaterial–human tissue interface [50]. There are two classic phenotypes of macrophages, which include either pro-inflammatory M1 and pro-healing (anti-inflammatory) M2 polarization, with the latter being responsible for tissue repair enhancement [51,52]. Implantation of biomaterial causes activation of M1 macrophages, and the chemoattractants and cytokines released in this phase stimulate osteoclastogenesis [53,54]. Moreover, activated M1 macrophages release cytokines with pro-inflammatory potential, which attract mesenchymal stem cells from local niches [55].

While M1 macrophages are vital in the initial phase of hydroxyapatite integration, an extended duration of M1 presence is responsible for chronic inflammation [56], causing higher expression of fibrous proteins as well as granuloma formation and encapsulation of an implant, which results in unsuccessful biomaterial implantation [57]. There are three subsets of M2 macrophages: M2a, M2b, and M2c [58]. While M2a and M2b are considered as mainly regulatory macrophages influencing Th2 lymphocytes, M2c is fundamental in tissue remodelling, suppression of inflammation, and promotion of angiogenesis [59].

The effective transition between M1 and M2 activation is responsible for balanced bone tissue regeneration. IL-10 and other anti-inflammatory cytokines are essential in providing an adequate microenvironment for osteogenesis [60]. 

Multiple studies have suggested the influence of biomaterial nanostructure on macrophage morphology [35,61,62]. 

### 2.3. Adhesive Cells Recruitment, Integrins, and Remodelling of the Cytoskeleton

Integrins belong to a family of cell surface receptors and mediate extra- and intracellular interactions [63]. They enable cell aggregation and direct migration and are composed of two subunits: alpha and beta [64]. Monocytes/macrophages express three types of beta subunit: B1, B2 and Beta3. Throughout B1, alfa4 and alfa5 bind to fibronectin and alfa 6 to laminin. Among B2, alfaL, alfaM and alfaD are specific for ICAM (intracellular adhesion molecules), alfaX attaches to fibrinogen, and C3bi complement fragment. alfaVB3 integrins attach to vitronectin [65]. The ability to adsorb proteins such as fibronectine or vitronectine to enable the further adhesion of blood-derived proteins is crucial in implanted biomaterial [66]. Due to increased amounts of attachment proteins, more osteoblasts and osteoblast precursors can potentially bind to the biomaterial which enhances bone ingrowth [67]. Moreover, these adhesive proteins on a HAp surface arrange a provisional matrix for further cell adhesion [68]. The deficiency of these proteins results in deteriorated attachment of bone-derived cells [69]. 

Subsequently, macrophages spread over the hydroxyapatite structure and undergo cytoskeleton remodelling [70]. The binding of proteins to the extracellular integrin’s domain activates its cytoplasmic domain that connects to intracellular particles [71]. Transduction of extracellular signals activates focal adhesion kinase (FAK) that regulates further focal adhesions and binds to cytoskeletal proteins such as paxillin [72]. Integrin receptors, FAK, as well as other kinases including ERK (extracellular signal-regulated kinase) and paxillin, talin, or vinculin interaction enables cytoskeleton remodelling [73,74].

### 2.4. Osteogenesis

Cell adhesion modulated by integrins triggers multiple intracellular cascades essential for cell destiny. Mitogen-activated protein kinase (MAPK) signalling activated by integrins in the process of osteogenesis currently enjoys great interest by scientists. Numerous studies reported on the role of the MAPK signalling pathway in modifying cell differentiation into osteoblasts [75,76,77,78].

Both major MAPK signals p38 and ERK play a crucial role in an indirect modulation of mesenchymal stem cell differentiation into an osteogenic lineage [79].

Osteogenesis in place of HAp implantation occurs most likely due to osteoconduction [21]. In this process, hydroxyapatite acts as a matrix for vascular proliferation where migrating proosteoblasts create neighbouring tissue [80]. In other terms, osteoconduction is the capability of bone growth on a biomaterial surface. Osteoinductive CaP-based ceramics such as hydroxyapatite also present a high affinity for multiple bone growth factors [81]. Calcium phosphates are known for their biocompatibility, whereas calcium ions are known to stimulate osteoblastic mechanisms through ERK1/2 and PI3K/Akt activation [75]. Phosphates modulate the growth and differentiation of osteoblasts via IGF-1 and ERK 1/2. They also enhance the expression of bone morphogenic protein (BMP) [82,83].

Osteoinduction means the ability to enhance progenitor cells to differentiate towards osteoblastic lineages [84]. Bone marrow-derived mesenchymal stem cells (BMSCs) are recruited from bone marrow to the non-osseous implant sites through blood circulation. This contributes to ectopic bone formation which is induced by osteoinductive CaP ceramics such as HAP [85]. Differentiation of BMCS requires the expression of pro-osteogenic genes such as Runt-related transcriptional factor 2 (Runx2) [86].

In a study conducted by Campi et al., nHAp (HAp nanoparticles) added to cell cultures caused enhanced synthesis of OPN (osteopontin), OCN (osteocalcin), ALP (alkaline phosphate), DCN (decorin), and COL–III (collagen III) [87]. Bone-specific ALP and COL-I are early markers of osteogenesis, and other proteins brand further stages [88] of osteopontin functions to stabilize the matrix [89].

## 3. Use of Hydroxyapatite in Spine Surgery

### 3.1. Anterior Cervical Discectomy and Fusion (ACDF)

The first historically documented clinical use of HAp in anterior cervical discectomy and fusion (ACDF) was noted by Koyama and Handa [90]. ACDF is a conventional technique of surgical treatment of post-traumatic and degenerative conditions of the cervical spine such as cervical spondylosis, especially degenerative disc disease. These cases may lead to spinal instability, chronic pain, radiculopathy, and myelopathy. Decompression of the neural structures and restoration of spinal stability, the foraminal area, disc space height, and spinal alignment are the main objectives of the ACDF.

#### 3.1.1. Types of grafts for ACDF

Due to the morbidity related to autologous iliac bone graft harvesting, which has been used for ACDF in the past, alternative graft materials for ACDF have been developed. The purpose of the graft is not only to fill space after the discectomy, it should also be a scaffold for new bone mass formation, which has to withstand mechanical stress [91]. Moreover, the ideal bone substitute should be as non-traumatic as possible, restore natural spine curvatures, provide sufficient stability, and maintain the integrity of the endplates of vertebral bodies [92]. Currently, there are many alternatives for autografts in ACDF, such as titanium mesh cages (TMCs) [93], polyetheretherketone (PEEK) cages [94], carbon-fibre cages [95] and nanohydroxyapatite/polyamide cages [38]. Additionally, hydroxyapatite formulations can be used as extenders for these grafts.

#### 3.1.2. Hydroxyapatite Properties in ACDF

Hydroxyapatite is characterized by great osteoconductive properties, which show it as a potential alternative to the autogenous bone graft. This material shows almost equivalent arthrodesis effects to autografts [96,97]. Additionally, HAp has some advantages compared with autologous bone graft. This material is characterised by excellent biocompatibility and does not induce a foreign body reaction [92]. The use of HAp implants also eliminates morbidity at the donor site following autogenous iliac bone grafting and provides a shorter operation time. It may reduce the time of postoperative treatment and hospitalization [92].

Because of the lack of osteoinductive properties of HAp, it cannot stimulate bone growth itself. Thus, the assertion of full contact HAp with cancellous bone is necessary [98]. Therefore, osteoinductive features of HAp increase after resecting endplates of adjacent vertebral bodies due to exposure of cancellous bone [99]. A single material that compromises osteoconductive, as well as osteoinductive features, has not yet been developed. Thus, for better osteoinductivity, many authors suggest adding osteoinductive materials to the hydroxyapatite implant such as demineralized bone matrix (DBM) [100,101,102].

In situ, the HAp graft undergoes slight and slow absorption, while maintaining primary compressive strength. This is important, as the mechanical properties, namely fracture strength and stiffness, of HAp composite materials are sensitive to variation in the concentration of Hap [103,104]. In particular, both strength and stiffness decrease with decreasing HAp concentration, reflecting a decreasing capacity for effective stress transfer from the matrix material to the HAp phase [103,104]. Within 2 months after implantation, osteoblasts and osteocytes from adjacent vertebral bodies migrate between the pores of the HAp implant and stimulate the formation of new bone, which creates a connection between vertebrae [38,91]. Many studies have described several sequelae after the use of porous HAp grafts alone for ACDF, including dislodgement (3–4% of cases) [91], loss of height or subsidence [98], the emergence of radiolucent stripe [98], and breakage or cracks of the implant (2% of cases) [98,105]. Therefore, HAp should not be used in grafts alone, but as one of the components of the graft for providing better osteoconductive properties.

#### 3.1.3. Nanohydroxyapatite Cages

Grafts manufactured from nanocrystals of hydroxyapatite can also be used in ACDF. The advantage of nanocrystalline HAp is the similarity of its crystal sizes to natural bone crystals [106,107]. This feature provides faster bony fusion and increases osteoblast proliferation [108]. Case series, described by Timothy et al., have demonstrated 100% bony fusion promotion after using HApN cages in ACDF without serious side effects. Moreover, HApN cages have shown a higher tolerance to torsion, shear, and compression forces when compared to other synthetic cages such as a PEEK [109].

#### 3.1.4. Nanohydroxyapatite/Polyamide 66 Cages

The n-HAp/PA66 is a novel composite consisting of nanohydroxyapatite and polyamide 66, which has been recently applied in ACDF. Due to the similarity of PA66’s structure to collagen, this polymer is biocompatible with many human cells [110]. Therefore, such a composite better imitates the structure of natural bone than HAp alone. It has demonstrated good biocompatibility, osteoconduction, and safety in severe reports [111,112]. Moreover, biomechanical studies have shown similar mechanical properties of n-HAp/PA66 to that of cortical bone, especially Young’s elastic modulus, which results in lower stress shielding and better bone fusion [94,113]. A retrospective study conducted by Zhang et al. [114] has shown that the n-HAp/PA66 cage achieved a similar fusion rate in two-level anterior cervical corpectomy and fusion (ACCF) and a lower rate of subsidence compared to a titanium mesh cage (TMC). Furthermore, failures of the graft are not common in the use of this material. The main disadvantage is the subsidence of the implant. Fortunately, the subsidence rate is not high and ranges from 2% to 10.6% [38,113,115,116,117,118]. Moreover, it is lower than that of the titanium mesh cage and is similar to PEEK cages [115,118]. Therefore, the n-HAp/PA66 cage has a great potential to be considered as a better alternative to PEEK cages and TMC to increase fusion rates and decrease the incidence of failures.

#### 3.1.5. Hydroxyapatite/PEEK Cages

Regarding outcomes following ACDF, PEEK cages have demonstrated similar results compared to autograft [94], although there are objections against its osteointegration as well as problems with radiographic assessment. Osteointegrative features can be improved by adding materials characterised by osteoconductive properties such as HAp crystals. A number of techniques have been applied to transform the theoretical osteoconductive features of HAp into spinal fusion systems—from the integration of HAp with autograft to synthetic intervertebral implants [119]. Popular applications of hydroxyapatite in intervertebral cervical cages include composite, cage filler, and coating.

Chin et al. [120] have evaluated the intervertebral cage composed of 80% PEEK and 20% calcium hydroxyapatite in ACDF, as shown in Table 1. In VAS and NDI scores, they have observed significant improvement in the HAp PEEK group. Moreover, the trend towards fusion has been observed in the HAp PEEK group earlier than in the control group (3–5 months vs. 8 months). Additionally, there were no significant complications during the 12-month postoperative follow-up. Therefore, HAp PEEK cages can be effectively and safely used in ACDF with better outcomes in comparison to the PEEK cages alone.

The hydroxyapatite also can be used as a filler within the PEEK cage. In a prospective randomized study conducted by Yi et al. [119], hydroxyapatite has been used as a PEEK cage filler to improve osteoconductive properties. They compared clinical results between a PEEK cage filled with the HAp/β-TCP mixture and a PEEK cage filled with a mixture of HAp and DBM (demineralized bone marrow). β-TCP and DBM were additionally applied to provide osteoinductive properties. Comparing these two cages, clinical outcomes and fusion rates were not statistically significantly different. 

### 3.2. Lumbar Spinal Fusion

Lumbar spinal fusion heavily relies on using autografts or allografts as a material used during surgery. Thus, they show to be the most effective way to achieve proper stabilization [39]. However, the still growing number of new materials accessible to use in this procedure creates an opportunity of lowering the negative effects of harvesting a bone graft by the usage of artificial graft material. 

#### 3.2.1. Hydroxyapatite with Beta-Tricalcium

Evaluation of hydroxyapatite and beta-tricalcium phosphate mixed with bone marrow aspirate as a bone graft substitute in reference to an autologous bone graft, showing that it can be successfully used [39]. It is suggested that this technique can be used instead of autologous grafts in lumbar fixation based on fusion rates and stability of achieved fixation.

Comparisons made between implants made out of 60TCP40HA and natural bone substituted in Sprague–Dawley rats showed a different nature of bone formation between two types of material used [40]. The natural bone resulted in more peripheral bony matter formation; however, the TCP/HAp composite resulted in a more centralized process of ossification. Analysis of both groups using micro-CT resulted in another interesting observation: the percent of bone volume in the fusion region after 4 weeks showed no difference; however, after 8 weeks, the volume of TCP/HAp was about twice compared to the natural bone substitute group, which can suggest greater efficiency of TCP/HAp composite in case of ossification [Table 2].

#### 3.2.2. Nanocrystalline Hydroxyapatite

Nanocrystalline hydroxyapatite used in lumbar fixation shows good results as used in arthrodesis compared to autograft mixed with BMA and iliac crest autograft, after 12 months [107]. This similarity also applies to multilevel stabilization. Robbins, Stephen, et al. also reported no complications related to the posterolateral graft mass and no symptomatic nonunions. Materials made out of nano-hydroxyapatite/polyamide-66 were shown to be a reliable manner of performing lumbar stabilization due to well-maintained disc height [41]. They provided a low chance of unsuccessful fusion, required no autologous bone harvesting, and showed relatively fewer postoperative morbidities, as seen in the donor region.

### 3.3. Pedicle Screw Fixation

Pedicle screw fixation (PSF) is regarded as the gold standard of treatment of spinal instability following traumas, degenerative changes, tumours, and deformities [121,122,123,124]. Despite many advantages of PSF [42,43], the application of this method does not exclude cases of pseudoarthrosis. Many reports have shown complications after PSF, such as loosening, pull-out, or breakage of screws [125,126,127]. To increase the rigidity of fixation, some factors are important, such as the surgical insertion technique, type of implant, augmentation method, and bone mineral density (BMD) [128]. In patients with decreased BMD, especially osteoporotic patients, there is an increased risk of screw loosening, nonunion, and back-off of the pedicle screw due to the poor mechanical properties of their bone [129,130]. Bisphosphonates and PTH, which are used in osteoporosis treatment, may prevent the mentioned complications through the increasing volume of bone substance around the screws [131]. The standard material used in PSF for improving anchoring strength is PMMA bone cement [132,133,134,135,136]. Such augmentation of titanium pedicle screws can decrease the risk of implant failures [137,138]. However, the use of PMMA causes some disadvantages, such as exothermic and toxic properties of this material or risk of cement leakage and extravasation [135,136,139,140]. Recently, different formulations of HAp have been evaluated regarding increasing fixation strength.

#### 3.3.1. HAp Screw Coating

One of them is the HAp coating, which has proven to be an efficient method of improving the bone–implant interface. In the study with a porcine osteoporotic model, Ohe et al. [42] proved that HAp-coated titanium pedicle screws provided strong fixation at the bone–implant interface. A study conducted by Yi et al. [141] on the human cadaveric model has proven that pull-out forces of Hap-coated screws in the insertion stage were a bit lower than those with PMMA bone cement. However, HAp stimulates bone growth in contrast to PMMA. Additionally, in a group of osteoporotic patients, HAp provided greater fixation strength than PMMA. Therefore, HAp could be a better clinical alternative. Liu et al. [43] proved on a sheep model that the addition of collagen and chondroitin sulphate (CS) to HAp coating presents better outcomes in new bone formation on the screw surface than coating with HAp alone. In such a composite, HAp prevents the oxidation of the screw surface and effectively adsorbs CS and collagen. Additionally, collagen promotes bone growth by interacting with progenitor cells, osteoblasts, and osteoclasts [43]. HAp coating as augmentation in PFS is a promising method of increasing the fixation strength of the screws. However, it should be evaluated clinically in further studies.

#### 3.3.2. HAp Sticks

HAp sticks are another formulation of HAp used in PSF. HAp, as a stick form, can be positioned at the target location without problems, distally to the screw. Moreover, in comparison to PMMA cement bone, there is no risk of material leakage. Shin et al. [142] evaluated the use of HAp sticks with PSF in patients with degenerative spine disease. They have observed that the additional use of HAp sticks increases the initial screw fixation strength in patients with osteoporosis. The effectiveness of HAp sticks also has been proven by many ex vivo and animal studies as well as clinical studies [141,143,144]. Therefore, HAp stick augmentation can reduce the frequency of the screw failure occurrence.

#### 3.3.3. HAp Granules

The latest research, conducted by Kanno et al. [145] on osteoporotic patients, has evaluated the use of HAp granules as augmentation with percutaneous pedicle screw fixation (PPFS). They inserted 50% porous HAp granules into the screw hole using a special device designed by themselves [146]. This study has shown that stability, pull-out strength, insertion torque, and resistance to cyclic loads of screws in the osteoporotic spine increase considerably after the addition of HAp granules. Moreover, at one-year follow-up, the incidence of screw loosening decreased.

## Figures and Tables

**Table 1:** Comparison of the hydroxyapatite cages with other cages used in ACDF.

Type of Cage	Material	Fusion Rate	Time to Achieve Solid Fusion	Subsidence Rate	Disadvantages
Autograft	Natural bone harvested from iliac bone	85–100% [102]	~6 months [96]	~0% [96]	morbidity at the donor site, increased blood loss, limited amount
Standard cages					
TMC Cage	Titanium	94–96% [113,118]	5–7 months [93]	From 4 to 22% [93,113,115]	difficulty in radiographic assessment, stress shielding effect [93,113]
PEEK Cage	Polyetheretherketone	88–100% [94]	7–8 months [120]	From 9.8% to 14.3% [115]	lack of osteointegration of the cage, difficulty in radiographic assessment [94]
Hydroxyapatite cages					
nHA/PA66 Cage	Nanohydroxyapatite infiltrating into polyamide 66	97%–98% [113,115,118]	-	From 2 to 10.6% [38,113,115,116,117,118]	difficult radiographic assessment of solid fusion, but easier compared with TMC [113]
Hydroxyapatite/PEEK Cage	Composite of 80% PEEK and 20% calcium hydroxyapatite	~100% [120]	3–5 months [120]	-	lack of clinical studies, difficulty in radiographic assessment

**Table 2:** Formulations of HAp used in spine surgery.

Procedure	HAp Formulation
Anterior Cervical Discectomy and Fusion	Nanohydroxyapatite
	Nanohydroxyapatite/polyamide 66 composite
	Hydroxyapatite/PEEK coating
	Hydroxyapatite/PEEK composite
Lumbar Spinal Fusion	Hydroxyapatite/beta-TCP
	Nanohydroxyapatite
Pedicle Screw Fixation	Hydroxyapatite screw coating
	Hydroxyapatite sticks
	Hydroxyapatite granules

## Data Availability

Not applicable.
